# Effects of *Moringa oleifera* in Two Independents Formulation and as Neuroprotective Agent Against Scopolamine-Induced Memory Impairment in Mice

**DOI:** 10.3389/fnut.2022.799127

**Published:** 2022-03-01

**Authors:** Wawaimuli Arozal, Emni Purwoningsih, Hee Jae Lee, Agian Jeffilano Barinda, Abdul Munim

**Affiliations:** ^1^Department of Pharmacology and Therapeutics, Faculty of Medicine, Universitas Indonesia, Jakarta, Indonesia; ^2^Doctoral Program in Biomedical Science, Faculty of Medicine, Universitas Indonesia, Jakarta, Indonesia; ^3^Faculty of Medicine, Universitas Muhammadiyah Sumatera Utara, Medan, Indonesia; ^4^Department of Pharmacology, School of Medicine, Kangwon National University, Chuncheon, South Korea; ^5^Metabolic disorder, Cardiovascular, and Aging Cluster, Faculty of Medicine, Indonesia Medical Education and Research Institute (IMERI), Universitas Indonesia, Jakarta, Indonesia; ^6^Department of Pharmacognosy-Phytochemistry, Faculty of Pharmacy, Universitas Indonesia, Depok, Indonesia

**Keywords:** *Moringa oleifera*, acetylcholine esterase (AChE), TrkB, memory impairment, nuclear factor kappa B (NF-κB), formulation

## Abstract

**Background:**

The cognitive deficit has frequently been found in the elderly population. Several studies have shown that every single part of *Moringa oleifera*, including leaves, roots, and seeds, has abundant micronutrients, such as flavonoids, which improve the neurobehavioral capacity. However, herb parts that display optimal neuropharmacological properties remain unknown.

**Objective:**

We investigate whether *M. oleifera* seed oil (MOO) or aqueous *M. oleifera* leaves extracts (MOEs) may ameliorate memory impairment in mice induced with scopolamine (Sco). Additionally, the phytochemical analyses of those two independent formulations were analyzed.

**Methods:**

In this study, 2 ml/kg body weight (BW) of MOO and 500 mg/kg BW of MOE were orally administered to the mice for 28 days, followed by intraperitoneal injection of Sco (1 mg/kg) at the day 22–28 to induce cognitive impairment in those mice.

**Results:**

The Sco group showed memory retention impairment represented by the Y-maze and novel object recognition tests, significant enhancement of acetylcholine esterase (AChE) activity in hippocampus tissue (*p* < 0.0001), and increased the level of total antioxidant capacity (TAOC) in serum. Interestingly, the Sco-induced memory defect was improved and completely blunted the AChE exacerbation in Sco+MOO-treated mice (*p* < 0.0001), although the TAOC level was comparable among the groups. Mechanistically, both tropomyosin receptor kinase B (TrkB), as a brain-derived neurotrophic factor-receptor, and nuclear factor-kappa-light-chain-enhancer of activated B cells (NF-κB) protein expressions were enhanced with the hippocampus isolated from the Sco group. Nonetheless, pretreatment with MOO only, but not with MOE, ameliorated the enhanced protein expression levels of TrkB and NF-κB (*p* < 0.05 and *p* = 0.09, respectively).

**Conclusion:**

Our data reveal that MOO is preferable to MOE as a neuroprotective as evidenced by improving memory impairment. This effect, at least in part, through inhibiting the AChE and NF-κB activities and modulating the TrkB expression level.

## Introduction

*Moringa oleifera* (Lam) or *M. oleifera* (MO), known as “Kelor” in Indonesia, is a member of the Moringaceae family, traditionally used for daily consumption and medical purposes in Asia including Indonesia (1, 2). Every part of this plant, such as leaves, seeds, pods, roots, stem, and bark, is formulated into a powder, suspension, oil for oral administration, and cream for topical application (3–5). Additionally, MO leaves have been commonly consumed as vegetables in daily life due to their high nutritional value (6).

Several previous studies have documented the beneficial effects of MO, including antioxidant and neuroprotective effects (7). Those effect activities may be linked with phytoconstituents activity in most of its parts (7). For instance, the leaves are rich sources of macro and micronutrients, including phytochemicals, such as tannins, sterols, saponins, terpenoids, phenolics, alkaloids, and flavonoids (8). Similarly, the seeds contain oleic acid (*Ben oil*), antibiotics called pterygospermin, fatty acids, such as linoleic acid, linolenic acid, and behenic acid, and phytochemicals, such as tannins, saponin, phenolics, phytate, flavonoids, terpenoids, and lectins (9). As far as we know, no particular study compares the pharmacological activity of different parts of MO.

The neuroprotective effects become an emerging issue in MO primarily derived from the leaves. MO administration ameliorated reactive oxygen species (ROS) exacerbation in cerebral ischemia (10). MO leaves extract (MOE) is used to treat dementia and improve spatial memory. Those extracts have been shown to decrease the acetylcholine esterase (AChE) activity, thereby improving cholinergic function and cognition (11). However, most of the studies used leaves as the herbal source, and few studies used MO oil derived from seed. One study reported that MO seed oil (MOO) had a beneficial effect in preventing the methotrexate-induced oxidative stress-mediated cerebral neurotoxicity and inflammation in the rodent model (12). To the best of our knowledge, limited studies compare the neuropharmacological activity of MO in different parts of plant and formulation.

It is well known that Sco induces cholinergic system and memory circuits dysregulation in the brain together with the reduction of the expression of cAMP-response element-binding protein (CREB) and brain-derived neurotrophic factor (BDNF) in the central nervous system. Therefore, this study sought to investigate whether MOO or MOE may improve memory impairment in mice induced by Sco and their phytochemical analysis and the underlying mechanism (13). In this study, the molecular mechanism was focused on antioxidant, anti-inflammation, cholinergic activity, and CREB pathway.

## Methods

### Plant Material

The MOE leaves powder was obtained from PT Javaplant (Solo, Indonesia). It was made by an aqueous extraction method and filled in with maltodrexin. The MOO was purchased from PT Kelorina (Medan, Indonesia). In brief, the fresh MO seeds were oven-dried for 7 days and were separately blended with a seed blender. Subsequently, the melted seeds were then independently poured into an oil extraction machine that separated the oil from the residue at both high temperature and pressure. Furthermore, the oil was obtained and kept at about 60–100°C under degraded pressure to maintain the constant temperature in the oil.

### Qualitative Phytochemical Screening of MOE

The MOE was tested to identify the alkaloids, flavonoids, saponins, anthraquinones, and tannins using various methods. The tests were conducted in triplicates to confirm reliable results. Briefly, the alkaloids identification was examined using a method reported by Sabri et al. (14). The flavonoids identification was based on a method described by Sharma and Sarin (15). The triterpenoid saponins identification was performed using the precipitation and foam test as described by Ashour et al. (16); anthraquinone identification was conducted using Borntrager's test (17), and lastly, tannins identification was performed using ferric chloride as described by Ugochuhwu et al. (18).

### Gas Chromatography-Mass Spectrometry Analysis of MOO

The gas chromatography-mass spectrometry (GC-MS) analysis was conducted at the National Instrumentation Center for Environmental Management (Seoul National University, Seoul, Republic of Korea). Briefly, MOO samples were redissolved and derivatized by the addition of 50 μl of 20 mg/ml methoxyamine hydrochloride in pyridine and incubated at 30°C for 90 min for oximation. To each sample, add 50 μl N,O-bis trimethylsilyl trifluoroacetamide, for trimethylsilylation derivatization, and 50 μl of fluoranthene (1,000 ug/ml in pyridine as an internal standard), and heat the mixture at 60°C for 30 min. Each 1.0 μl aliquot of derivatized sample was injected in 40:1 split ratio into a Thermo scientific (Trace 1310/ISQ LT) GC-MS equipped with DB-5MS capillary column (60 m × 0.25 mm × 0.25 μm, Agilent J&W Scientific, USA). Helium was used as the carrier gas with a constant flow rate of 1.5 ml/min. The temperature program was as follows: the initial temperature of 50°C was maintained for 2 min, raised to 180°C at a rate of 5°C/min and held for 8 min, and increased to 210°C at a rate of 2.5°C/min, increased to 325°C at a rate of 5°C/min, and was maintained for 10 min. The temperature of the injector, transfer line, and ion source was set to 300, 310, and 270°C, respectively. The mass range (35–650 m/z) in a full-scan mode for electron impact ionization (70 eV) was applied. The solvent delay time was set to 14 min.

The identification of each metabolite was positively confirmed by comparison of retention time and mass spectral data with those of a NIST/EPA/NIH Mass Spectral Library (version 2.0 d, National Institute of Standards and Technology, Gaithersburg, USA). All metabolites were identified by comparing mass fragments with the standard mass spectra on the commercial database NIST with a similarity of more than 70%. The calculated area of each compound was normalized by dividing the internal compound (fluoranthene) peak area to give the semi-quantitative composition of components. Each compound was quantified against the internal standard by integrating peak areas.

### Animal Study

Twenty male BALB/c mice, 3 months old, and weighing between 40 and 45 g, were obtained from the animal house of Biopharma Laboratory and Animal Breeding, Bandung, Indonesia. In this study, we used male mice since they have higher anxiety levels and are more vulnerable compared with female mice in some established behavioral tests (19, 20). All these animals were kept in the closed system cages at the Animal Research Facility, Institute of Medical and Research, Faculty of Medicine Universitas Indonesia, Jakarta, Indonesia. All animals were housed in an environment with a constant temperature of about 25°C and a 12-h light/12-h darkness cycle, stress free, water *ad libitum*, and standard diet (consisting of 7% simple sugars, 4% fat, 4% fibers, 20% protein, 50% polysaccharide, and some micronutrients). The mice were acclimatized to the laboratory environment for 1 week. All experiments were carried out in accordance with internationally recognized principles for the use and care of laboratory animals and have been approved by the local ethics committee of the Faculty of Medicine of Universitas Indonesia with the reference number KET-1405/UN2.F/ETIK/PPM.00.02/2020.

### Experimental Procedures

The animals were divided into four groups which contained five mice in each group, as follows: normal group received distilled water (orally) and intra peritoneal (i.p.) normal saline throughout the experiment; Sco group received distilled water orally throughout the experiment and Sco (1 mg/kg i.p.) for 7 days (at the day 22 until 28); Sco+MOE group received MOE at the dose of 500 mg/kg body weight (BW) followed by the injection of Sco (1 mg/kg i.p.) for 7 days; and Sco+MOO group received MOO at the dose of 2 ml/kg BW followed by the injection of Sco (1 mg/kg i.p.) for 7 days. The dose of MOO (21–23) and MOE (24) was chosen based on the previous publications. MOE and MOE were administrated orally for 28 consecutive days and 30 min before Sco injection. The dose and the duration of administration of Sco, MOO, and MOE were mentioned based on a previous report (25–27). The Sco working solution (Sigma-Aldrich, St. Louis, MO, United States) was prepared using 0.9% NaCl. The MOE solution was prepared using distilled water. The mice were introduced one after the other into the novel object recognition (NOR) on days 29 and 30, and Y-maze was carried out on day 30. The NOR test and Y-maze test were done separately, in the different animal experiments. Immediately after the behavior test, mice were sacrificed, and the hippocampus and plasma were collected for further biochemical and immunoblotting analysis. The blood was collected through the cardiac puncture, and the whole brains were harvested, then rinsed with ice-cold buffer saline to exclude the remaining blood, followed by hippocampus resection. The plasma was separated by centrifugation at 3,000 g for 5 min at 4°C.

Moreover, the aliquots of plasma were stored at −80°C for further analysis. The hippocampus was homogenized in ice-cold buffer saline for AChE activity or cold radioimmunoprecipitation buffer for immunoblotting analysis. The homogenates were then centrifuged at 10,000 *g* to separate nuclear debris, and the protein concentration was measured.

### Behavioral Study (Y-maze Test)

The effects of the drugs on the spontaneous alternation behavior of mice were measured for 8 min and recorded using an automated system. The test was performed by the method described by Gil Yong Lee et al. (28). The spontaneous alternation (%) was derived from the total number of alternations divided by the total number of arm entries minus two, which was multiplied by 100 as shown in the following equation: % Alternation = [(Number of alternations)/(Total number of arm entries−2)] × 100.

### Behavioral Study (NOR Test)

To evaluate the non-spatial learning of object identity of mice, which relies on the multiple brain region, we performed the NOR test. In the NOR task, the apparatus consisted of an open field box with the size of 40 cm × 40 cm × 40 cm and made of white acrylic material. This test was performed by the method described by Denninger et al. (30) Furthermore, 1 day prior to the experiment, each mouse was habituated to the open field box without any object for 6 min. On the experiment day, each mouse was placed in the open field for 10 min and allowed to freely explore the two identical objects. After an interval of 20 min post training session, one of the old objects use was substituted into a new object and the mouse was left to explore the objects for 10 min. The time spent with each object was recorded. An analysis of video recorded was performed by two independent and blinded experimenters. The percentage of investigation time of novel object was calculated using the formula [TB/(TA+TB) × 100], where TA and TB are time spent exploring familiar object (A) and novel object (B), respectively (30).

### Acetylcholine Esterase Activity Analysis

The activity of AChE was carried out using a mouse AChE ELISA Kit from Sigma (MAK119) according to the manufacturer's instructions. This assay is an optimized version of the Ellman method in which thiocholine, produced by AChE, reacts with 5,5'-dithiobis (2-nitrobenzoic acid) to form a colorimetric (412 nm) product, proportional to AChE activity present. One unit of AChE is the amount of enzyme that catalyzes the production of 1.0 μM of thiocholine per minute at room temperature at pH 7.5.

### Total Antioxidant Capacity Analysis

Total antioxidant capacity (TAC) in the serum was determined spectrophotometrically using a commercial kit from Sigma (MAK187) according to the manufacturer's instructions. The absorbance was measured at 570 nm.

### Immunoblotting

Protein extraction derived from hippocampus tissue using RIPA buffer added with protease and phosphatase inhibitors (Sigma P0044; Sigma P8340). The protein concentration was equalized using a Coomassie Plus (Bradford) commercial assay kit (Merck B6916) spectrophotometrically at a wavelength of 595 nm (31). The protein isolation was then stored at −80°C for further analysis. The protein samples were separated by their molecular weight by sodium dodecyl sulfate–polyacrylamide gel electrophoresis (SDS-PAGE); after that, they were transferred to a nitrocellulose membrane. Membranes were incubated with 5% skim milk in TBS-T for 30 min and labeled with the first antibody diluted in blocking buffer overnight at 4°C, followed by keeping in secondary antibody diluted in blocking buffer. The signals were then detected using the Chemiluminescence Alliance 4.7 (Uvitec) with enhanced chemiluminescence substrate (BioRad). Finally, the specific target proteins' band was visualized using the Gel Documentation, quantified, and normalized with glyceraldehyde 3-phosphate dehydrogenase (GAPDH) expression levels with the data presented in arbitrary units.

All antibodies for Western blot analysis were purchased from Cell Signaling Technology (Beverly, MA). GAPDH (14C10), Rabbit mAb (CST#2118), NF-κB p65(D14E12), rabbit mAb (CST#8242), CREB (48H2) Rabbit mAb (CST #9197), p-CREB (Ser 133) (87G3) Rabbit mAb (CST#9198) and TrkB (C80E3) Rabbit mAb (CST #4603, and Anti Rabbit IgG HRP Link-Antibody (CST #7074S).

### Statistical Analysis

Data were presented as the ±SEM. These data were analyzed using the one-way ANOVA followed by a Tukey's test. The *p*-values of ^*^p < 0.05, ^**^p < 0.01, ^***^p < 0.001, and ^****^p < 0.0001 were considered as statistically significant. All statistical analyses were performed with SPSS version 26. All graphs were presented in GraphPad Prism version 9.0.0.

## Results

### Phytochemical Analyses of MOE and MOO

Phytochemical qualitative analysis of MOE isolated from various organic solvents detected the active compounds of triterpenoid, polyphenolic, saponin, and tannin flavonoid. However, anthraquinone and alkaloid were barely found in this herb.

**Figure 1:**
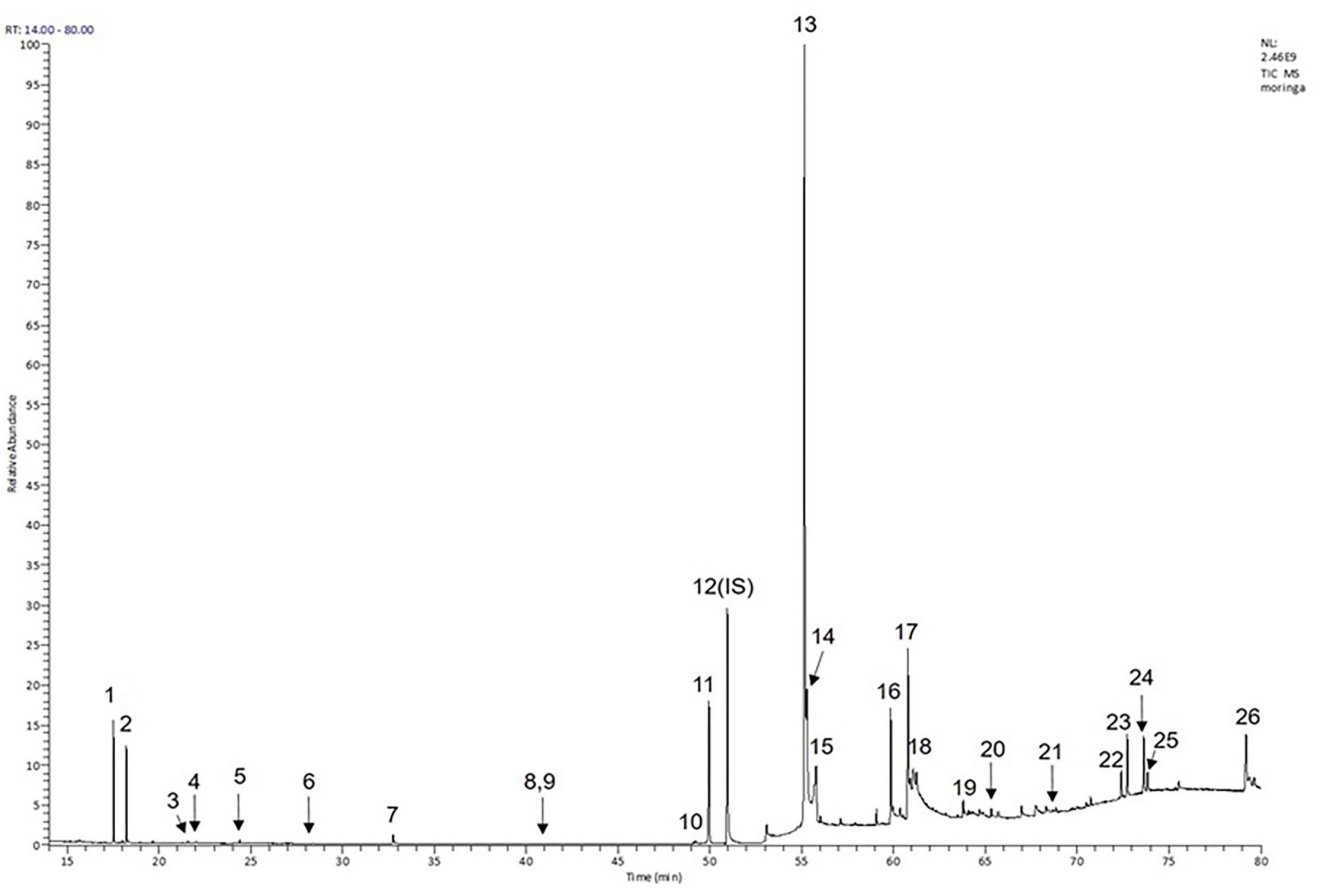
Chromatogram obtained from the gas chromatography-mass spectrometry (GC-MS) with the *Moringa oleifera* seed oil (MOO).

Moreover, the GC-MS analysis in MOO showed 26 active phytochemical compounds, represented from the twenty six peaks (Figure 1). The details of the active component name, including their retention time (RT) and concentration (peak area %), are presented in Table 1. Among those compounds, palmitic acid, oleic acid, stearic acid, stigmasterol, and β sitosterol showed the highest percentages among the compounds identified by GC-MS.

**Table 1:** Gas chromatography-mass spectrometry (GC-MS) analysis of *Moringa oleifera* seed oil.

**No**	**Nature of compound**	**Retention time (min)**	**Name of compound**	**Molecular formula**	**Molecular weight (g/mol)**	**Peak area (%)**
1	Unidentified	17.52	Unidentified	-	-	2.356
2	Unidentified	18.24	Unidentified	-	-	3.433
3	The aromatic carboxylic acid (-COOH)	21.59	Benzoic acid 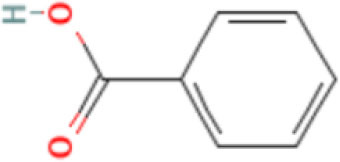	C_7_H_6_O_2_	122.12	0.141
4	Sugar alcohols	22.06	Glycerol 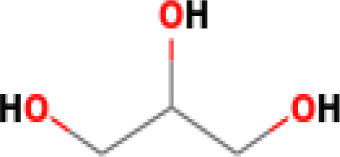	C_3_H_8_O_3_	92.09	0.074
5	Unidentified	24.42	Unidentified	-	-	0.206
6	Unidentified	28.41	Unidentified	-	-	0.012
7	Fatty acids	32.77	Lauric acid 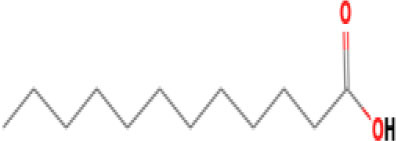	C_12_H_24_O_2_	200.32	0.431
8	Unidentified	40.97	Unidentified	-	-	0.015
9	Fatty acids	41.47	Myristic acid 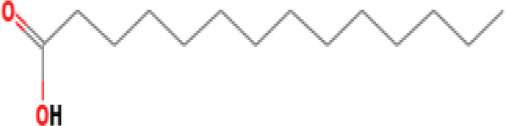	C_14_H_28_O_2_	228.37	0.028
10	Fatty acids	49.14	Palmitoleic acid 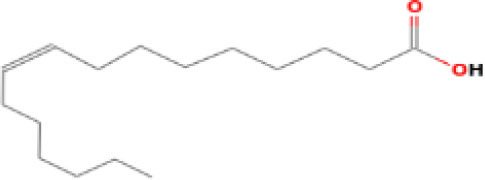	C_16_H_30_O_2_	254.40	0.051
11	Fatty acids	49.96	Palmitic acid 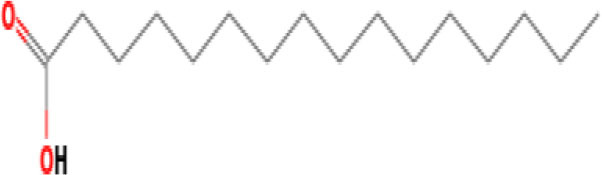	C_16_H_32_O_2_	256.42	6.569
12	Polycyclic aromatic hydrocarbons	50.96	Fluoranthene 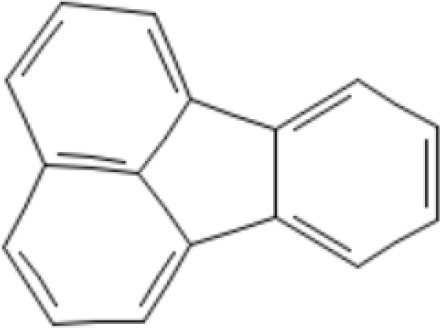	C_16_H_10_	202.25	33.088
13	Fatty acids	55.15	Oleic acid 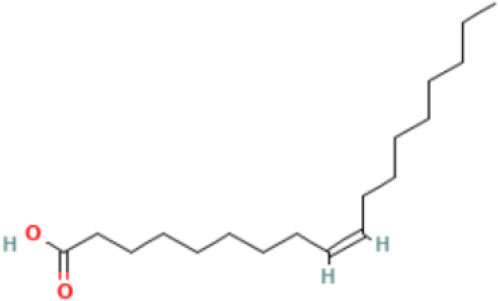	C_18_H_34_O_2_	282.5	18.925
14	Fatty acids	55.28	Oleic acid 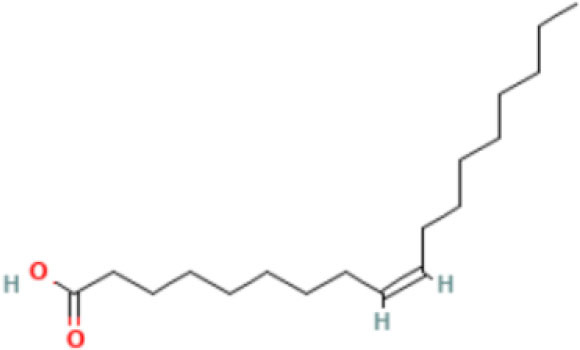	C_18_H_34_O_2_	282.5	18.925
15	Fatty acids	55.77	Stearic acid 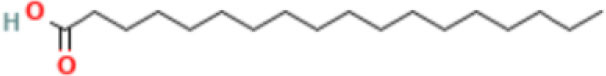	C_18_H_36_0_2_	284.5	2.815
16	Unidentified	59.85	Unidentified	-	-	2.354
17	Unidentified	60.78	Unidentified	-	-	3.886
18	Unidentified	61.24	Unidentified	-	-	0.293
19	Unidentified	63.81	Unidentified	-	-	0.898
20	Glycerides	65.33	1-Monooleoylglycerol 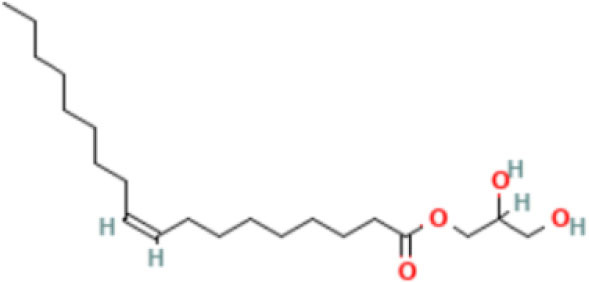	C_21_H_40_O_4_	356.5	0.156
21	Vitamin E	68.83	Tocopherol-γ-tms-derivative 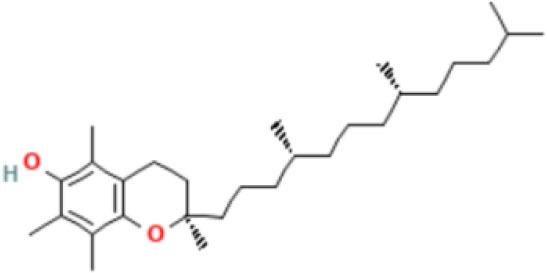	C_31_H_56_O_2_Si	488.8	0.106
22	Phytosterols	72.39	Campesterol 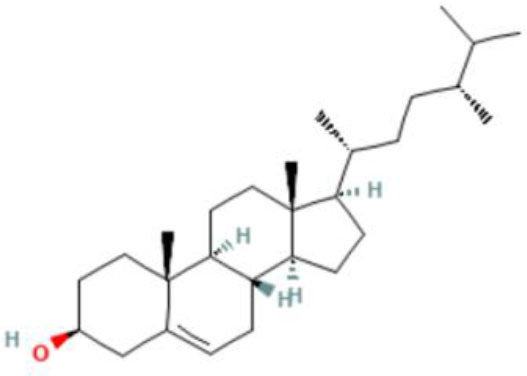	C_28_H_48_o	400.7	0.590
23	Phytosterols	72.73	Stigmasterol 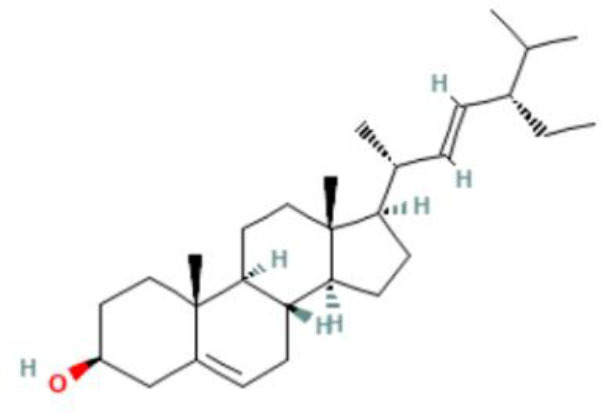	C_29_H_48_o	412.7	1.378
24	Phytosterols	73.61	β-Sitosterol 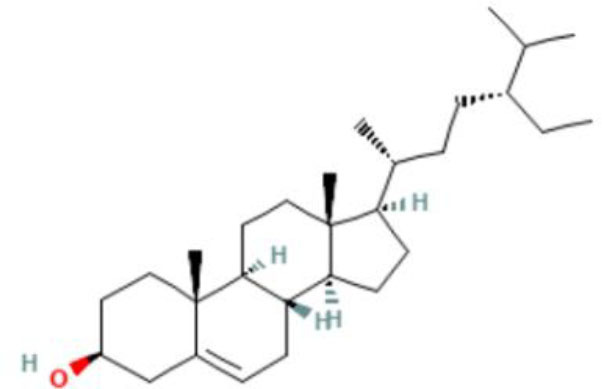	C_29_H_50_O	414.7	1.198
25	Unidentified	73.83	Unidentified			0.291
26	Unidentified	79.21	Unidentified			1.785

*The 3D (picture) name of compound were captured from pubchem.ncbi.nlm.nih.gov*.

### The Effects of MOE and MOO in the Memory Impairment Caused by Sco Treatment

**Figure 2:**
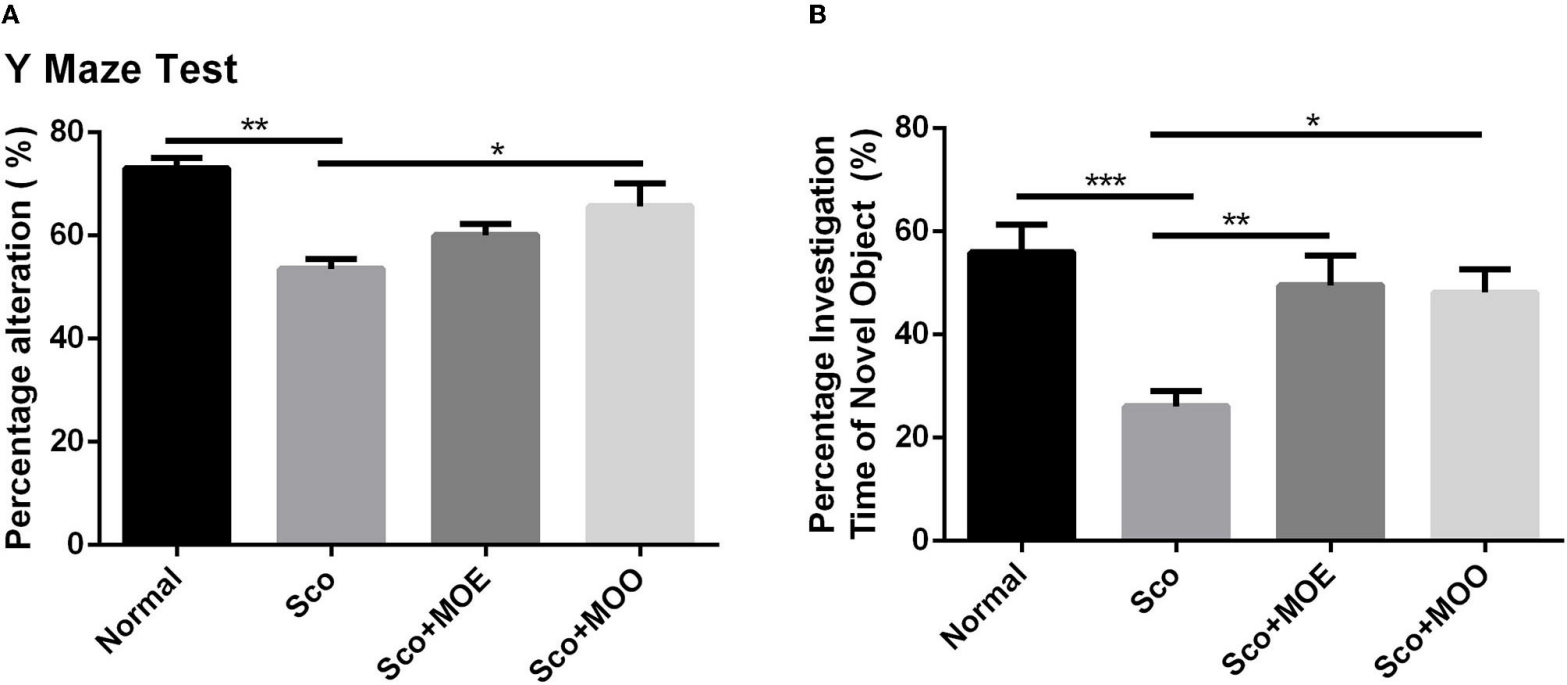
**(A)** Effects of *M. oleifera* leaves extract (MOE) and MOO on scopolamine (Sco)-induced memory impairments in Y-maze test by assessed the percentage alteration. Each column represents mean ± SEM of six mice. Data analysis was performed using one way ANOVA, followed by Tukey's multi comparison test. **p < 0.01; *p < 0.05. MOE, aqueous leaves exctract of *M.oleifera*; MOO, *M.oleifera* seed oil. **(B)** Effect of MOE and MOO on Sco-induced memory impairments in novel object recognition (NOR) test by assessed the percentage investigation time to novel object. Each column represent mean ± SEM of six mice. Data analysis was performed using one way ANOVA, followed by Tukey's multi comparison test. ***p < 0.001; **p < 0.01; *p < 0.05. MOE, aqueous leaves exctract of *M.oleifera*; MOO, *M.oleifera* seed oil.

We initially performed the spatial and recognition memory impairment in mice by administrating Sco i.p. (32). Of note, Sco injection in Sco, Sco+MOE, and Sco+MOO groups for 7 consecutive days exhibited a robust spatial recognition memory deterioration compared with the normal group in the Y-maze test (Figure 2A). Similarly, the NOR test showed a significant reduction in the percentage of investigation time of novel object compared with the normal group (*p* < 0.0001) (Figure 2B). Interestingly, Sco-induced recognition memory deficit was effectively restored by oral administration of MOO (1 ml/kg BW) in parallel with the increased percentage of spontaneous alteration significantly compared with the Sco group (*p* < 0.05), but this effect did not occur in the MOE treatment. The MOE-treated group showed a tendency to increase the percentage of spontaneous alteration was not statistically significant. Meanwhile, the NOR test showed a significant increase in the percentage of investigation time of the novel object in both MOE and MOO treatments compared with the Sco group (*p* < 0.01 and *p* < 0.05, respectively).

### The Effects of MOE and MOO in the AChE Activity in Hippocampus Tissue

**Figure 3:**
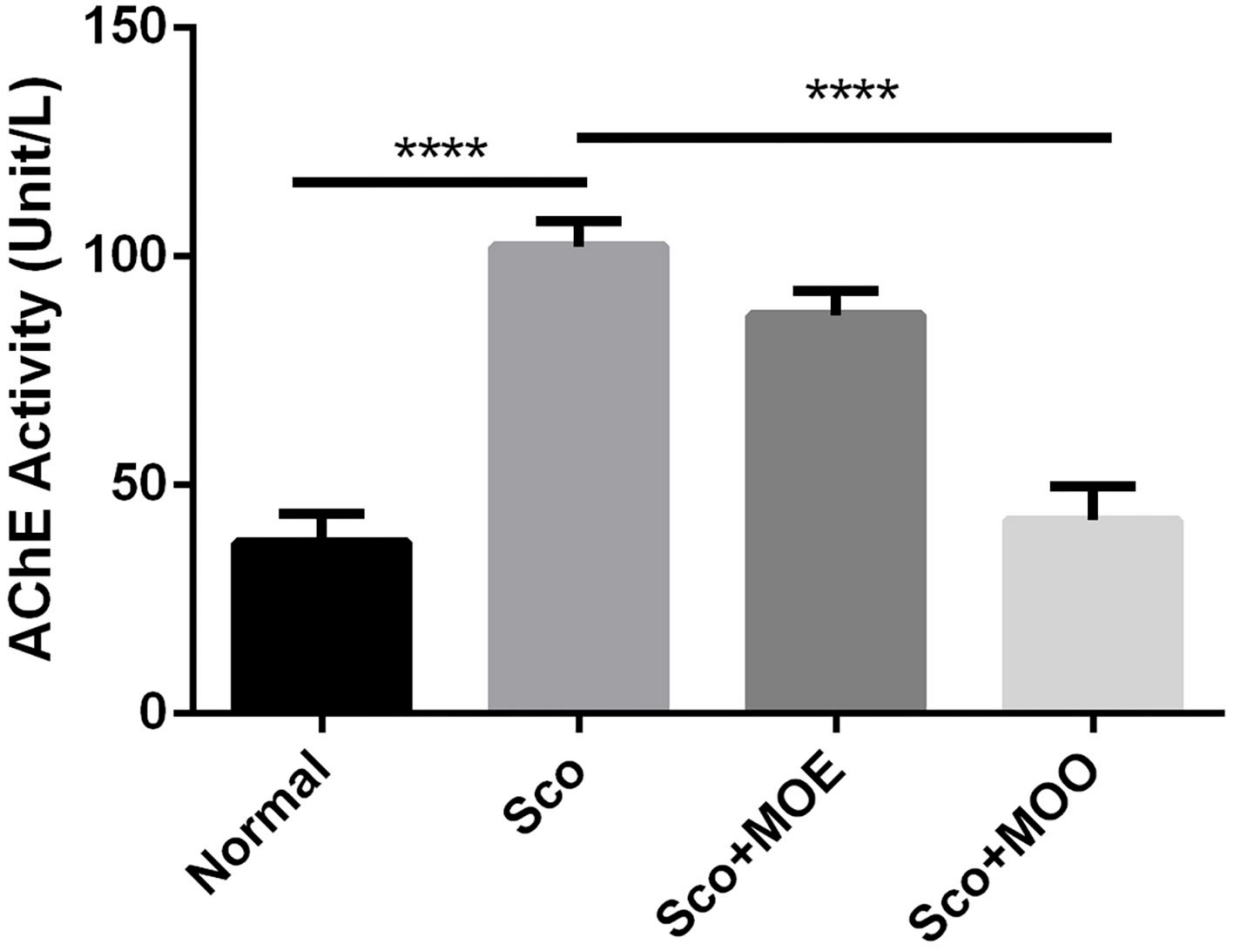
Effects of MOE and MOO on Sco-induced memory impairments in the activity of acethylcholine esterase. Each column represents mean ± SEM of six mice. Data analysis was performed using one way ANOVA, followed by Tukey's multi comparison test. ****p < 0.0001. MOE, aqueous leaves exctract of *M.oleifera*; MOO, *M.oleifera* seed oil.

To further investigate the mechanism of MO in both MOE and MOO, we analyzed the AChE activity which represented cholinergic status in mild cognitive impairment (33). The mice treated with Sco only significantly increased the AChE activity compared with the normal group (*p* < 0.0001). Moreover, pretreatment with MOO, but not with MOE, showed completely blunted the Sco-mediated AChE exacerbation (*p* < 0.0001) (Figure 3).

### The Effects of MOE and MOO on TAC Level in Serum

**Figure 4:**
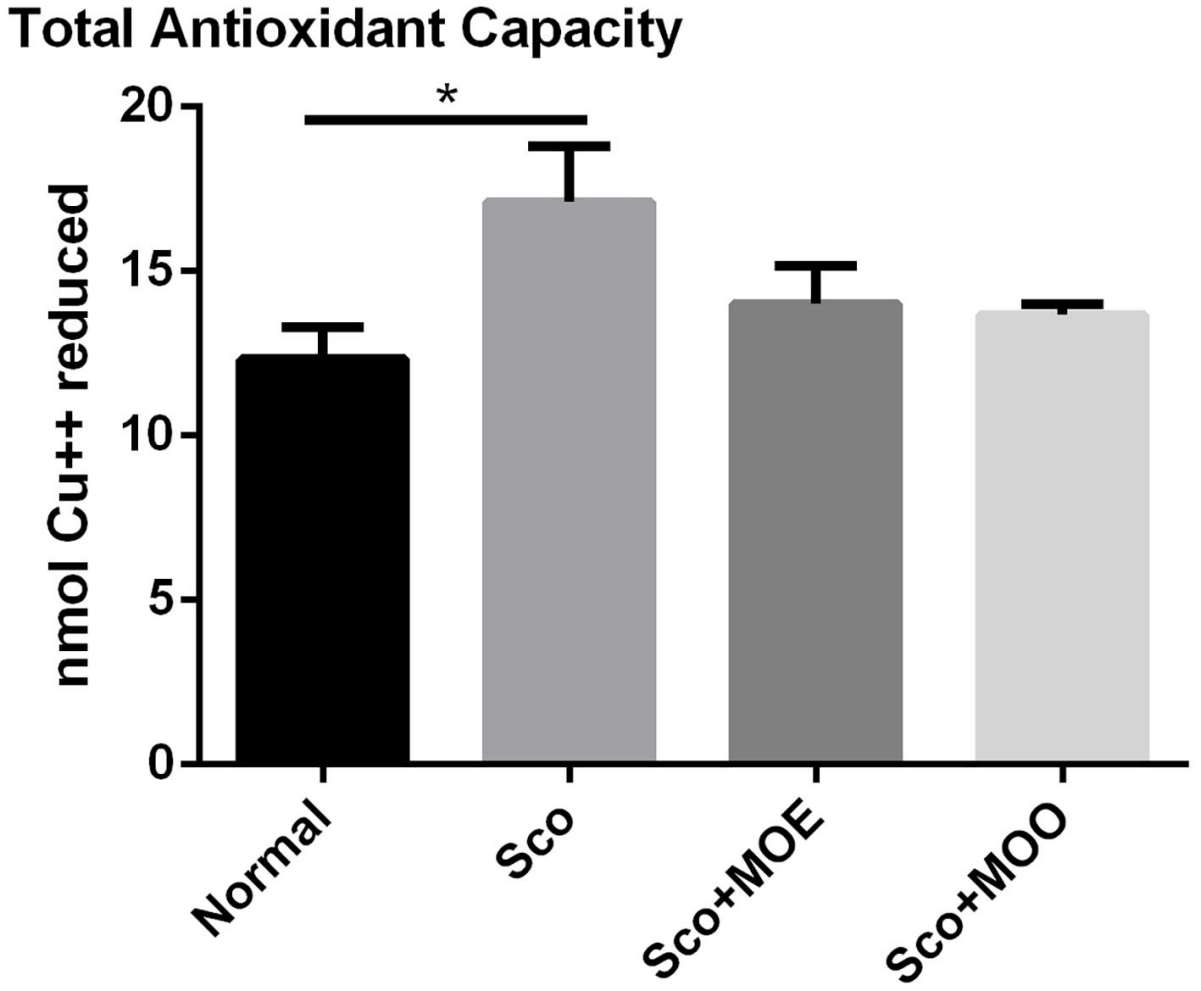
Effects of MOE and MOO on Sco-induced memory impairment in total antioxidant capacity (TAC) in serum. Each column represents mean ± SEM of six mice. Data analysis was performed using one way ANOVA, followed by Tukey's multi comparison test. *p < 0.05. MOE, aqueous leaves exctract of *M.oleifera*; MOO, *M.oleifera* seed oil.

Oxidative stress in the brain is exacerbating cognitive impairment (34). Therefore, to determine whether the antioxidant mechanism plays a pivotal role in the neuroprotective action of MO, the TAC level from circulating serum was determined as an oxidative stress marker (35). Similarly, the Sco group showed a significant increase in the TAC level in serum compared with the normal group (*p* < 0.05). However, pretreatment with either MOE or MOO failed to show ameliorated effect of TAC level (Figure 4).

### The Effects of MOE and MOO on the Expression of NF-κB

**Figure 5:**
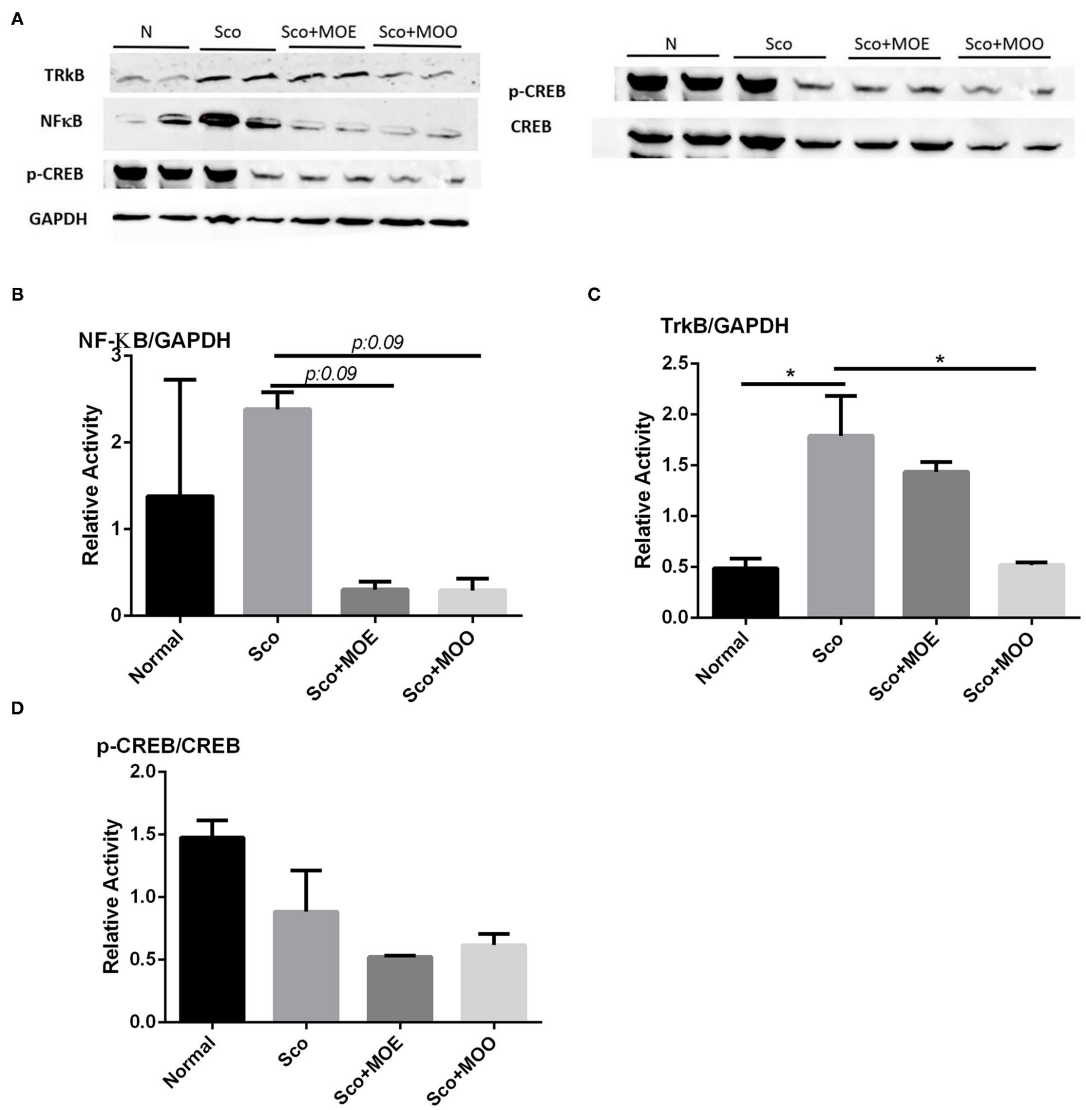
**(A)** Representative western blot gel in the hippocampus tissue of tropomyosin receptor kinase B (TrkB), nuclear factor-kappa-light-chain-enhancer of activated B cells (NF-κB), p- cAMP-response element-binding (CREB), CREB, and glyceraldehyde 3-phosphate dehydrogenase (GAPDH). **(B)** Quantified band analysis of NF-κB protein expression. Quantified result were normalized to GAPDH expression. Each column represents mean ± SEM of six mice. Data analysis was performed using one way ANOVA, followed by Tukey's multi comparison test. MOE, aqueous leaves exctract of *M.oleifera*; MOO, *M.oleifera* seed oil. **(C)** Quantified band analysis of TrkB protein expression. Quantified result were normalized to GAPDH expression. Each column represents mean ± SEM of six mice. *p < 0.05. MOE, aqueous leaves exctract of *M.oleifera*; MOO, *M.oleifera* seed oil. **(D)** Quantified band analysis of p-CREB protein expression. Quantified result were normalized to CREB expression. Each column represents mean ± SEM of six mice. MOE, aqueous leaves exctract of *M.oleifera*; MOO, *M.oleifera* seed oil.

Nuclear factor kappa-light-chain-enhancer of activated B cells (NF-κB) is highly contributed to cognitive impairment as a master regulation of pro-inflammatory pathways in the brain (36, 37). The hippocampus isolated from the Sco group revealed the highest NF-κB expression at the protein level (even though the quantification of the protein level expression was not statistically significant), while pretreatment with MOE and MOO tends to prevent the enhancement of this expression (*p* = 0.09) (Figures 5A,B).

### The Effects of MOE and MOO on the Expressions of TrkB, CREB, and Phosphorylated CREB

To explore whether MOE and MOO improve the neurogenesis in the hippocampus, the receptor of BDNF, namely, tropomyocin receptor kinase B (TrkB) expressions level were examined at the protein level as the downregulation of BDNF as one of the responsible neurotrophic factors for neurogenesis (38). Mice treated with Sco alone exhibited significant upregulation of TrkB protein expression compared with the normal group (*p* < 0.05); in contrast, pretreatment with MOO detected decreased expression level compared with those in Sco group only (*p* < 0.05) (Figures 5A,C). Moreover, we investigated the CREB signaling pathway in this study. Though we could not find any significant finding among the groups (Figures 5A,D).

## Discussion

In this study, we revealed the distinct memory-enhancing effects of MOE or MOO in the Sco-induced memory impairment mice model. To the best of our knowledge, this is the first report which compared the neuroprotective effect of two independent formulations of *M. oleifera* and highlighted the involvement of oxidative stress parameter, cholinergic activity, inflammatory, and neurogenesis markers in the Sco-induced memory impairment rodent model.

Phytochemical screening analyses identified several active compounds in MOE, such as triterpenoid, polyphenolic, saponin, tannin, and flavonoid, as the major secondary metabolite, which is consistent with the previous study (29). Of note, the flavonoids, such as quercetin and rutin, and polyphenolic found in MOE are known to display antioxidant and neuroprotective properties (7). However, we did not further analyze the details of flavonoids in this extract and considered this issue as a limitation of this study. In the GC-MS analysis, a total of 26 volatile compounds were found in MOO, such as palmitic acid, oleic acid, stearic acid, stigmasterol, β sitosterol, and tocopherol. These compounds have previously been isolated from other medicinal plant species and were believed to play an essential role in their pharmacological activity (39). In line with our study, a study had demonstrated that MOO was abundant in some primary bioactive agents, including unsaturated fatty acids, such as oleic acids, β-Sitosterol, and stigmasterol (9), which were believed to have neuroprotection effect (40).

In the present study, we performed Y-maze and NOR tasks to understand the MOE and MOO effects in regulating spatial and recognition memory. The Y-maze test relies on the innate investigative nature of mice to explore a new environment to assess the short-term spatial memory, while NOR is an established behavioral test to assess the recognition memory (41). Our results showed that mice treated with Sco only impaired the short-term and recognition memory, as evidenced by reducing the percentage of spontaneous alteration and investigation time of the novel object. This result was supported by another report that found the Sco exposure reduced spontaneous alternation percentage in the Y-maze test (28) due to interference with acetylcholine function at the synapse system and partly increased acetylcholine esterase activity in the cortex and hippocampus (42). Moreover, cholinergic nervous system degeneration-mediated acetylcholine degradation has known to be one of the most important causes of memory impairment (43). In our study, MOO pretreatment has a more beneficial effect compared with MOE in improving spatial and recognition memory. MOO effectively improved Sco-induced impairment in the Y-maze and NOR tests in parallel with significantly suppressed the Sco-induced increase AChE activity in the hippocampus tissue (Figures 2A,B, 3). This result suggests that MOO ameliorated short-term and recognition memory loss, possibly through acetylcholine system restoration. In addition, the richness of fatty acid, benzene, fatty aldehydes, terpenoids, tocopherol, and stigmasterol in MOO could explain the neuroprotective effect of this oil. Similarly, other reports confirmed that MOO could be an effective neuropharmacological drug also for amnesia by the modulation of cholinergic activity (44).

Oxidative stress is a major cause of neurodegenerative disease; thus, this inhibition is considered a promising strategy to prevent neurodegenerative disease. Ample evidence showed the potent antioxidant effect of *M. oleifera* in most of the parts of this plant (45), as previously described also elsewhere in human neuroblastoma (SH-SY5Y cell) (46). In this study, the TAC level in serum, which represented oxidative status in the brain, was analyzed; and found that both MOO and MOE failed to ameliorate the increased TAC serum levels in mice treated with Sco. Further study is needed to perform the oxidative status directly in the hippocampus tissue, for example, by checking the Nrf2 signaling pathway which is an archetypical regulator of the antioxidant defense enzymes (47).

A previous study showed that MO has potentially attenuated NF-κB signaling in lead-induced cortical brain toxicity (48). NF-κB has been implicated in synaptic plasticity, learning, and memory abilities (49). Sco causes NF-κB mediated inflammation causing cholinergic nerve damage and cognitive deficiency (50). Hence, we sought that MO may protect Sco-induced memory deficit, at least due to its anti-inflammatory properties (51). In the current study, we found that mice treated with Sco only increased NF-κB expression at the protein level and MOO pretreatment, but not MOE, for 28 days tend to ameliorate this protein expression in parallel with its ability to improve the activity of AChE. Future studies will be interesting to analyze whether MOO is also involved in the downstream target of NF-κB.

A BDNF neurotrophic factor is an important gene to improve memory function and neurogenesis through the CREB signaling activation. BDNF is also involved in neuronal survival and further activating CREB after binding to its receptor tyrosine kinase B (TrkB) (52). A previous study reported an interconnection between Trk B and NFκB. An exogenous BDNF activates TrkB receptors leading to the activation of NF-kB (53). A previous study showed that *M. oleifera* seed extracts improved the BDNF level together with CREB signaling activation in Sco-induced learning and memory impairment mice. However, this study did not investigate the TrkB involvement (44). Therefore, we further evaluate the effects of MOO and MOE on TrkB activation and the CREB signaling pathway. Mice treated with Sco alone exhibited significant upregulation of TrkB expression level, and MOO pretreatment markedly ameliorated this protein expression. This result suggests that MOO may modulate the TrkB through the downregulation of NF-κB transcription factors. However, in the present study, we did not observe any effect of MOO and MOE in the CREB signaling pathway. Further research is needed to clarify this phenomenon.

In conclusion, our data demonstrated that MOO was preferable to MOE to improve the short-term memory impairment induced by Sco, partly mediated by cholinergic activation and inhibiting NF-κB protein expression level, thereby reducing the upregulation of TrkB as a BDNF receptor. We sought that the insufficient neuroprotective effect of MOE might be due to the less distribution of this formulation into the brain through the blood-brain barrier compared with the MOO formulation (54). The amount of active compound in each formulation achieved in the organ targets, such as the brain should be investigated in further research.

## Data Availability Statement

The original contributions presented in the study are included in the article/supplementary material, further inquiries can be directed to the corresponding author/s.

## Ethics Statement

The animal study was reviewed and approved by Komite Etik Penelitian Kesehatan Faculty of Medicine of Universitas Indonesia.

## Author Contributions

WA, HL, AB, and AM designed the experiment, analyzed the data, and wrote the manuscript. WA, EP, HL, and AB experimented and contributed to the data analysis. All authors contributed to the article and approved the final submitted version.

## Funding

This research was supported by grants from HIBAH PUTI DOKTOR 2020, Universitas Indonesia, Indonesia (NKB-573/UN.2.RST/HKP.05.00/2020), and Kangwon National University (2019), Republic of Korea.

## Conflict of Interest

The authors declare that the research was conducted in the absence of any commercial or financial relationships that could be construed as a potential conflict of interest.

## Publisher's Note

All claims expressed in this article are solely those of the authors and do not necessarily represent those of their affiliated organizations, or those of the publisher, the editors and the reviewers. Any product that may be evaluated in this article, or claim that may be made by its manufacturer, is not guaranteed or endorsed by the publisher.
